# Building data science capacity in the public health workforce: a Public Health Data Science Life Cycle framework

**DOI:** 10.3389/fpubh.2026.1887767

**Published:** 2026-07-17

**Authors:** Emily M. Burke, Jordan A. Fox, Sarah Gusman, Kaitlin Tager, Rebecca Wurtz, Gulzar H. Shah, Maureen Y. Lichtveld

**Affiliations:** 1 Association of Schools and Programs of Public Health, Washington, DC, United States; 2 School of Public Health, University of Minnesota, Minneapolis, MN, United States; 3 Jiann-Ping Hsu College of Public Health, Georgia Southern University, Statesboro, GA, United States; 4 School of Public Health, University of Pittsburgh, Pittsburgh, PA, United States

**Keywords:** core competencies, data modernization, data science framework, public health data science, workforce development and training

## Abstract

The digital transformation of public health systems has increased the need for a workforce capable of using data science to inform population health decision-making. However, public health workforce development efforts have not fully integrated data science competencies into training and professional development pathways. This paper presents a competency mapping framework, and a proposed national training agenda designed to operationalize data science skills for the public health workforce. Drawing on and synthesizing existing public health competency frameworks, accreditation standards, and governmental public health workforce task analyses, competencies were mapped to an eight-stage Public Health Data Science Life Cycle. Competency gaps were then identified, and additional competencies were developed to address emerging needs in public health data science practice. The analysis found that existing competencies are concentrated in the middle stages of the Public Health Data Science Life Cycle – particularly data collection and management, data analysis and modeling, and data interpretation and implications – with fewer competencies addressing earlier stages, such as problem framing, and later stages, including communication and life cycle preservation. Key gaps were identified in areas including project feasibility and problem definition, data governance and interoperability, bias and ethical data use, data interpretation and visualization, and the communication and translation of findings to inform policy and community action. Building on these findings, this paper proposes a practical framework to guide the integration of public health data science competencies into workforce training and continuing professional learning. By aligning competencies with the full life cycle of public health data science practice, this approach supports a more comprehensive and applied model for workforce development, with the goal of strengthening data-informed decision-making and improving population health outcomes.

## 1 Introduction

Information and knowledge systems are a core component of public health and, together with workforce capacity and organizational systems, form a pivotal part of public health infrastructure (1). The practice of public health is increasingly shaped by the availability of large-scale digital data and advanced analytic technologies (2). Public health agencies – at the federal, state, Tribal, local, and territorial levels – rely on complex information systems to conduct surveillance, evaluate programs, and respond to emerging health threats (3). At the same time, advances in artificial intelligence, machine learning, natural language processing, and computational modeling have expanded practitioners’ ability to generate actionable insights from both structured and unstructured data (4). As a result, proficiency in public health data science – and the ability to interpret analytic results – are becoming essential for effective practice (5).

As public health becomes more data-intensive, the role of data science in public health practice has become increasingly central – it integrates methods from statistics, computer science, epidemiology, and informatics to analyze complex health data and inform evidence-based decision-making (2). In this context, data science within the field of public health can be defined as “the study of formulating and rigorously answering questions in order to advance health and well-being using a data-centric process that emphasizes clarity, reproducibility, effective communication, and ethical practices” (2). This definition encompasses activities across the data life cycle, including hypothesis generation, study design, data collection and management, analysis, dissemination, and translation, and reflects a perspective shaped by advances in computational methods, reproducibility, and interdisciplinary collaboration (2). These approaches rely on digital tools and data systems to collect, manage, analyze, and interpret health data, positioning data science as a central component of modern public health. In turn, data science enables the translation of data into actionable insights that inform policy and improve health outcomes (6, 7).

Despite these advances, the integration of data-driven approaches into public health practice remains uneven. Workforce shortages, limited training opportunities, and insufficient integration of public health data science competencies into workforce training represent major barriers to modernization (8, 9). The COVID-19 pandemic further exposed gaps in workforce capacity to manage complex data systems and interpret real-time health information, underscoring the need to strengthen data science skills across the public health workforce (8).

In response to these challenges, competency frameworks have emerged as important tools for guiding workforce development and training. However, few frameworks explicitly organize competencies across the full life cycle of public health data science practice. To address this gap, this paper presents a competency mapping framework that identifies existing public health data science competencies, reveals areas of limited coverage, and defines additional competencies to address emerging needs. These competencies are organized within an eight-stage Public Health Data Science Life Cycle to support a more comprehensive and applied approach to workforce development.

## 2 Conceptual framework: the Public Health Data Science Life Cycle

Data science involves a sequence of activities requiring distinct digital skills and technologies. To organize competencies across these processes, the authors adapted a Public Health Data Science Life Cycle framework informed by established models of data science workflows (10).

The Public Health Data Science Life Cycle was developed through an iterative process involving multiple rounds of review and refinement by an expert panel composed of academic and practice-based public health leaders with expertise in public health workforce development, governmental public health practice, epidemiology, informatics, and data science. The expert panel included 13 academic and practice-based public health leaders representing schools and programs of public health, governmental public health agencies, and national public health organizations.

The framework was designed to organize competencies across the progression of public health data science activities, from identifying and framing public health problems through the collection, analysis, interpretation, communication, and preservation of data and findings. The eight stages of the framework were selected because they collectively capture the major functions required to translate data into actionable public health knowledge and practice.

Framework refinement occurred through iterative discussion and expert review. The Public Health Data Science Life Cycle framework was developed through expert panel review and refinement, while the competency mapping and gap analysis were conducted by the study team using the framework as an organizing structure. The resulting Public Health Data Science Life Cycle serves as an organizing structure for competency mapping and workforce development rather than as a prescriptive model of practice.

## 3 Analytic approach

This analysis uses a structured competency mapping approach to examine existing public health competencies relevant to public health data science. The authors reviewed public health competency frameworks, accreditation criteria, and job task analyses to identify previously vetted public health competencies.

Key source documents included:

- Council on Education for Public Health 2021 Accreditation Criteria (11)
- Adapting and Aligning Public Health Strategic Skills (de Beaumont Foundation) (12)
- Core Competencies for Public Health Professionals (Public Health Foundation, Council on Linkages Between Academia and Public Health Practice) (13)
- National Board of Public Health Examiners 2016 Public Health Job Task Analysis (14)
- 10 Essential Public Health Services (Public Health Accreditation Board, Public Health National Center for Innovation, and the de Beaumont Foundation) (15)
- Public Health Accreditation Board Standards and Measures (16)
- The Essential Public Health Functions in the Americas (Pan American Health Organization) (17)

Each source was reviewed using targeted keyword searches for terms, including *data science, data collection, data analysis, data analytics, informatics, data management,* and *surveillance.* Identified competencies were extracted and mapped to the eight stages of the Public Health Data Science Life Cycle. Competency extraction, mapping, and synthesis were conducted by members of the study team. Where competencies could reasonably apply to multiple stages, placement was determined through iterative review and discussion among the authors based on the competency’s principal intent and application. Following competency mapping, competencies were aggregated within life cycle stages and domains to examine the distribution of competency coverage across the framework.

The authors then conducted a gap analysis to identify areas where competencies were absent, underrepresented, or insufficiently specified relative to contemporary public health data science practice. Competency gaps were identified through comparison of mapped competencies across life cycle stages and through expert review of functions considered essential to contemporary public health data science practice. Proposed competencies were developed to address these gaps and aligned with the corresponding life cycle stage.

This analysis focuses on competencies relevant to the governmental public health workforce in the United States, including practitioners at the federal, state, Tribal, local, and territorial levels.

## 4 Results

The competency mapping revealed that data science skills are required across all stages of the Public Health Data Science Life Cycle. While existing competency frameworks emphasize analytic and epidemiologic methods which map to the middle stages of the life cycle, fewer competencies address earlier stages, such as problem framing, and later stages, such as communication, decision-making, and life cycle preservation. Competency gaps were identified in domains related to data governance, interoperability, ethical use of data, and translation of findings into policy and practice. This suggests that current public health training may not provide practitioners with the data science skills needed in practice.

### 4.1 Competencies identified across the Public Health Data Science Life Cycle

Competencies derived from established public health competency frameworks were identified and mapped across the eight stages of the Public Health Data Science Life Cycle. Competencies from multiple frameworks are organized by life cycle stage, and further grouped into associated domains (e.g., “Identify the problem,” “Research methods,” “Data collection methods”).

Using this mapped structure, competencies were extracted and aggregated to quantify their distribution across life cycle stages and domains. A total of 79 competencies were identified. Table 1 summarizes the number of competencies associated with each stage of the life cycle and corresponding domains.

**Table 1:** Distribution of identified public health competencies across the Public Health Data Science Life Cycle.

Public health data science life cycle stage	Public health data science domain(s)	Number of competencies
Problem framing	Problem identification	7
Research design	Research methods; Evaluation and assessment	14
Data collection, management, and surveillance	Data collection methods; Data management and sharing; Surveillance	23
Data analysis and modeling	Programming and statistical methods; Quantitative and qualitative data analysis	14
Data interpretation and implications	Quantitative and qualitative data interpretation; Data visualization and interpretation	13
Communication and dissemination of methods, results, and interpretation	Scientific communication and dissemination	2
Decision-making and action	Data-informed decision-making	6
Preservation of life cycle	Documentation	0

As shown in Table 1, competencies were unevenly distributed across the eight stages of the Public Health Data Science Life Cycle. The largest number of competencies were identified in data collection, management, and surveillance (*n* = 23), followed by research design (*n* = 14), data analysis and modeling (*n* = 14), and data interpretation and implications (*n* = 13). In contrast, fewer competencies were identified in problem framing (*n* = 7) and decision-making and action (*n* = 6), while communication and dissemination (*n* = 2) and preservation of the data life cycle (*n* = 0) were minimally or not represented. This distribution highlights a relative emphasis on mid-stage analytic activities compared to earlier problem definition and later application and communication.

### 4.2 Identified competency gaps

Building on the competency mapping, gaps were identified by examining areas where competencies were absent, underrepresented, or insufficiently specified across the Public Health Data Science Life Cycle. This analysis considered both the presence of competencies within each domain and the extent to which they addressed key functions required for data-informed public health practice.

Across the life cycle, competency gaps were not evenly distributed. While some domains included well-established competencies, others demonstrated limited coverage or lacked competencies altogether in critical areas. In particular, gaps were identified in domains related to communication, data governance and stewardship, and the translation of data into policy and practice. These gaps reflect areas that are less emphasized in existing public health competency frameworks, which tend to focus more heavily on technical and analytic skills than on application, communication, and life cycle continuity.

These findings are summarized in Table 2, which presents the distribution of identified competency gaps across life cycle stages and domains.

**Table 2:** Distribution of identified competency gaps across the Public Health Data Science Life Cycle.

Public health data science life cycle stage	Public health data science domain(s)	Number of competency gaps
Problem framing	Problem identification	2
Research design	Research methods; Evaluation and assessment	2
Data collection, management, and surveillance	Data collection methods; Data management and sharing; Surveillance	5
Data analysis and modeling	Programming and statistical methods; Quantitative and qualitative data analysis	1
Data interpretation and implications	Quantitative and qualitative data interpretation; Data visualization and interpretation	4
Communication and dissemination of methods, results, and interpretation	Scientific communication and dissemination	8
Decision-making and action	Data-informed decision-making	1
Preservation of life cycle	Documentation	5

As shown in Table 2, competency gaps were most pronounced in communication and dissemination (*n* = 8), followed by data collection, management, and surveillance (*n* = 5) and preservation of the data life cycle (*n* = 5). Additional gaps were identified in data interpretation and implications (*n* = 4), while fewer gaps were observed in problem framing (*n* = 2) and research design (*n* = 2). Minimal gaps were identified in data analysis and modeling (*n* = 1) and decision-making and action (*n* = 1).

The results reinforce the imbalance observed in the distribution of competencies, with mid-life cycle technical areas more fully developed, while earlier and later stages remain underrepresented. In particular, the concentration of gaps in communication and dissemination highlights limited emphasis on translating complex data into meaningful, actionable insights for diverse audiences. Similarly, gaps in the preservation of the data life cycle stage point to insufficient attention to data stewardship, documentation, and long-term data use – critical components for sustaining data-informed public health practice.

Taken together, the analysis indicates that existing competency frameworks do not fully support the application of data science across all stages of public health practice, particularly in areas related to communication and the translation of findings into policy and practice. To address the identified gaps, additional competencies were developed and aligned with each stage of the Public Health Data Science Life Cycle (Table 3).

**Table 3:** New and expanded public health data science competencies aligned with the Public Health Data Science Life Cycle.

Life cycle stage	New or expanded competencies
Problem framing	Determine the significance and justification of a project Evaluate the feasibility of a project, including its risks, value, and resource needs
Research design	Define data elements needed to answer research questions Collaborate with subject and process matter experts to develop research protocol
Data collection, management, and surveillance	Develop methods and tools for collecting data Secure permission for data collection or access Adhere to human subject protection requirements Ensure compliance with regulations regarding privacy, security, and confidentiality Apply standard definitions and data structures to enhance system and data interoperability
Data analysis and modeling	Address inaccuracies posed by bias in data collection and interpretation
Data interpretation and implications	Interpret results at multiple levels Use data to inform decision-making and policy development Visualize data using tools and technologies Apply design principles to reveal patterns, trends, and correlations
Communication and dissemination of methods, results, and interpretation	Synthesize findings in clear, concise writing that provides meaningful context Describe the strengths and limitations of data sources, analysis, and applications Communicate findings using narrative structure and visual design principles to inform decision-making Explain the implications for policy impact of health and wellbeing Discuss the impact of the findings on communities and populations Prioritize communication of results to research participants and affected populations Outline methodological and other changes desirable for future studies on the topic Present results, interpretations, and recommendations in venues tailored to target audiences
Decision-making and action	Apply results, recommendations, and evaluation data to plan the next stage of inquiry, research, or action
Preservation of life cycle	Document research methods, data artifacts, and governance records Apply results, recommendations, and evaluation data to guide the next phase of investigation, research, or action Maintain data to ensure integrity and minimize loss Store data safely to prevent accidental loss Record data backup policies

## 5 Discussion

### 5.1 Implications for public health workforce development

Building on the competency mapping and the Public Health Data Science Life Cycle framework, the proposed national training agenda supports the development of data science competencies across the public health workforce. Central to this approach is the need to expand capacity in data governance, privacy, and the ethical use of data, ensuring that public health practitioners are equipped to manage complex data systems. Integrating data science competencies across core public health curricula ensures that these skills are treated as foundational rather than specialized or optional components of training for public health practice.

The proposed national training agenda also highlights the importance of developing skills in data interpretation and communication, enabling practitioners to translate findings into meaningful insights for diverse audiences. Strengthening collaboration between public health and data science disciplines is another key priority, as effective public health practice requires the integration of technical expertise with domain-specific knowledge, such as partnerships between epidemiologists and data scientists, collaboration with informatics specialists to design and manage data systems, and engagement with policymakers and community stakeholders to ensure that analytic findings are relevant and actionable.

Rather than serving as a prescriptive set of competencies, the proposed national training agenda provides a practical framework to guide the selection and development of data science workforce training. Organized across the Public Health Data Science Life Cycle, it outlines the types of learning objectives and skill areas that organizations should prioritize when vetting training opportunities for staff development.

Across the life cycle, training programs should enable public health practitioners to perform key functions required for data-informed practice. Training recommendations were developed by translating mapped competencies and identified gaps into applied learning objectives aligned with each stage of the Public Health Data Science Life Cycle.

#### 5.1.1 Problem framing

Training should prepare staff to assess community health status and determinants, conduct health assessments, identify emerging threats, and evaluate the feasibility, significance, and equity implications of proposed interventions. Effective training in this area should support the development of clear, actionable public health questions grounded in real-world context.

#### 5.1.2 Research design

In this stage, training should enable professionals to access, select, and synthesize data from multiple sources and apply appropriate qualitative, quantitative, mixed-methods, and policy analysis approaches. It should also support the definition of relevant data elements and the design of rigorous, context-specific research or evaluation projects in collaboration with subject matter experts. In addition, training should build capacity to select and apply appropriate evaluation methods, assess community health status, analyze health system changes and their impacts, and use surveillance data and continuous quality improvement approaches to strengthen public health policies, programs, and functions.

#### 5.1.3 Data collection, management, and surveillance

Training in this stage should prepare staff to ethically design and implement methods for collecting valid and reliable quantitative and qualitative data while adhering to human subjects protections and securing appropriate permissions. It should support ethical and secure data management, including compliance with privacy and confidentiality requirements, the use of standard definitions and interoperable data structures, and the implementation of data management plans. Training should further address the use of surveillance systems, including the ability to manage, interpret, and communicate data to monitor health risks, guide public health action, and evaluate the effectiveness and limitations of surveillance tools.

#### 5.1.4 Data analysis and modeling

Training programs should enable practitioners to analyze quantitative and qualitative data using statistical methods, software, and informatics tools, applying both descriptive and inferential techniques to support evidence-based decision-making.

#### 5.1.5 Data interpretation and implications

In this area, training should prepare practitioners to analyze and interpret data using statistical and informatics tools, apply data visualization techniques and visual design principles, and identify patterns and trends. Trainings should also prepare staff to draw conclusions to inform policy and practice, identify drivers of health disparities, and assess the strengths and limitations of data sources, research findings, and surveillance systems.

#### 5.1.6 Communication and dissemination

Effective training should enable professionals to apply and communicate research and evaluation findings using qualitative, quantitative, mixed-methods, and policy analysis approaches. This includes assessing strengths, limitations, and implications, and translating results through clear, context-rich writing, visual design, and audience-specific communication strategies to inform decision-making, policy, and community impact.

#### 5.1.7 Decision-making and action

Training should prepare staff to guide equitable, evidence-based decisions, advocate for data-driven policies and programs, and plan subsequent research, action, or evaluation.

#### 5.1.8 Preservation of the data life cycle

In this stage, training should support the ability to document research methods and data governance processes, preserve and back up data to prevent loss, and use findings to inform future research, evaluation, and public health action.

Taken together, this life cycle-aligned training approach provides a practical and scalable tool for workforce development by helping organizations identify training opportunities that build the full range of skills required for public health data science practice. Aligning training selection with the stages of the life cycle supports a more comprehensive and consistent approach to workforce development and helps ensure that practitioners are prepared to apply data-driven approaches across the full continuum of public health practice.

### 5.2 Practical application of framework

Grounding training in the Public Health Data Science Life Cycle framework enables public health practitioners to develop technical skills and the capacity to apply data-informed approaches to complex health challenges. Practitioners must be able to define clear, actionable public health questions; ensure data quality and ethical use; apply appropriate analytical methods; and translate findings into actionable insights that inform decision-making and improve health outcomes.

Advancing this vision requires a tiered, action-oriented approach that builds workforce capacity through leadership engagement, integration of data science into routine practice, and opportunities for applied learning. Progress begins with fostering leadership commitment and organizational readiness. Leaders and champions play a critical role in articulating the value of data science for advancing public health priorities. Early investments, including targeted funding, academic partnerships, and alignment with public health data modernization efforts, can help establish a foundation for building data science capacity within the public health workforce. Initial activities may include leadership briefings, introductory training on data science concepts, and efforts to build a shared understanding of how data can be used to support decision-making and program improvement.

Embedding public health data science competencies into workforce training and organizational processes is essential for sustaining and scaling data science capacity within the public health workforce. Institutions and public health jurisdictions can begin by focusing on a small number of priority areas, such as incorporating non-surveillance data sources or applying analytics to program planning and expanding over time. Integration should also extend to workforce planning, including job descriptions and professional development, as well as to partnerships with academic institutions through guest instruction, internships, and joint projects.

Workforce capacity is strengthened when practitioners apply public health data science skills to their own data and programmatic contexts. Hands-on learning opportunities, mentorship, micro-credentials, and peer learning communities can support continued skill development and confidence. Success should be assessed not only by the acquisition of technical skills, but also by the extent to which data science is integrated into program decision-making, strengthens evaluation practices, and contributes to improved population health outcomes.

Strengthening public health data science capacity is an ongoing, multi-level process that requires coordinated action across national, institutional, and instructional levels. At the national level, alignment of funding and policy with data modernization and workforce development goals is critical. Establishing shared standards for practice, supporting collaboration between academic and practice-based organizations, and sustaining investment in training pathways, including degree and certificate programs, internships, and reskilling initiatives, can help ensure long-term impact.

### 5.3 Contribution to the public health data science literature

Existing public health competency frameworks, including those developed by the Council on Linkages (13) and the Council on Education for Public Health (11), provide a strong foundation for workforce development, but do not organize competencies around data science for public health. The framework proposed in this paper extends these efforts by integrating data science competencies across the stages of public health practice, from problem definition through data-driven decision-making and system preservation.

Building on these contributions, this work advances the growing literature on public health data science workforce development by operationalizing public health competencies within a data science life cycle framework that connects analytic processes to public health practice. This life cycle perspective is important in the context of ongoing digital transformation, which is reshaping how public health systems collect, analyze, and use data (2). While data science tools offer powerful capabilities for analyzing complex health data, they also raise important ethical and governance challenges (18, 19). Issues related to privacy, data security, and algorithmic bias highlight the need for training programs that integrate ethical frameworks alongside technical competencies (7, 20).

A key strength of this paper is the deliberate use of a life cycle-based framing. Using a life cycle approach extends traditional competency models by connecting skills across all stages of public health practice, from problem definition to data preservation. This integrated perspective supports workforce development by ensuring that competencies are applied to real-world decision-making and system improvement.

### 5.4 Limitations and future directions

This study has several limitations. First, the competency mapping process relied on expert review and synthesis of existing competency frameworks, accreditation standards, and workforce analyses rather than empirical validation of competencies in practice. As a result, the framework represents a conceptual synthesis and organizing structure for workforce development rather than a validated competency model. The framework should therefore be interpreted as a workforce development tool rather than a validated model of competency acquisition or workforce performance.

Second, competency assignment to Public Health Data Science Life Cycle stages required expert judgment. Although competencies were reviewed iteratively by the authors, formal inter-rater reliability measures were not assessed. Different reviewers may have categorized some competencies differently across life cycle stages.

Third, the source frameworks used in this analysis were selected to support workforce development within the United States governmental public health system. Consequently, the resulting framework may not fully reflect competency needs in clinical settings, private-sector organizations, or public health systems in other countries, including low- and middle-income countries. Accordingly, the transferability of the framework to international public health systems should not be assumed and warrants future study.

Finally, the proposed national training agenda has not yet been evaluated in workforce training or educational settings. Future research should examine implementation of the framework, assess its usefulness for training design and workforce development, and evaluate its impact on practitioner competencies, organizational capacity, and public health outcomes.

## 6 Conclusion

Preparing the public health workforce for the digital age requires a systematic approach to defining and operationalizing data science competencies (2). The competency mapping framework presented in this paper offers a practical model for integrating data science skills into public health workforce training. By aligning competencies with the life cycle of public health data science practice, this framework supports the development of a workforce capable of leveraging digital technologies to improve population health and advance health equity.
